# TCR Transgenic Mice: A Valuable Tool for Studying Viral Immunopathogenesis Mechanisms

**DOI:** 10.3390/ijms21249690

**Published:** 2020-12-18

**Authors:** Yong-Bin Cho, In-Gu Lee, Yong-Hyun Joo, So-Hee Hong, Young-Jin Seo

**Affiliations:** 1 Department of Life Science, Chung-Ang University, Seoul 06974, Korea; dydqls1218@cau.ac.kr (Y.-B.C.); izac1995@cau.ac.kr (I.-G.L.); dozen95@cau.ac.kr (Y.-H.J.); 2 Department of Biotechnology, The Catholic University of Korea, Bucheon 14662, Korea

**Keywords:** TCR transgenic mice, immunopathogenesis, viral infection, T cell

## Abstract

Viral infectious diseases are a significant burden on public health and the global economy, and new viral threats emerge continuously. Since CD4^+^ and CD8^+^ T cell responses are essential to eliminating viruses, it is important to understand the underlying mechanisms of anti-viral T cell-mediated immunopathogenesis during viral infections. Remarkable progress in transgenic (Tg) techniques has enabled scientists to more readily understand the mechanisms of viral pathogenesis. T cell receptor (TCR) Tg mice are extremely useful in studying T cell-mediated immune responses because the majority of T cells in these mice express specific TCRs for partner antigens. In this review, we discuss the important studies utilizing TCR Tg mice to unveil underlying mechanisms of T cell-mediated immunopathogenesis during viral infections.

## 1. Introduction

Despite advancements in vaccine and antiviral drug development, viral infections still cause significant morbidity and mortality in humans. In addition to the recent emergence of Zika virus, MERS-CoV, and SARS-CoV-2, viruses including influenza virus, hepatitis C virus (HCV), and human immunodeficiency virus (HIV) continue to threaten human health. To counter these diseases, a fundamental understanding of viral pathogenesis is critical. Experimental animal models are widely used to evaluate the efficacy and safety of potential vaccines and antivirals. The mouse model is one of the most advantageous models because the mouse immune system is evolutionarily similar to that of humans. It is also low-cost, easy to genetically regulate, and genetically identical species can be readily obtained [1,2,3].

Since anti-viral T cell responses are critical to eliminating viruses and protecting against recurrent infection, intensive and in-depth analyses of T cell responses are essential in understanding immunopathological responses against viral infections. However, due to large T cell receptor (TCR) diversity (1 × 10^13^), complete and precise analyses of viral antigen-specific T-cell responses are almost impossible. To overcome this limitation, transgenic (Tg) mice with high frequencies of particular TCRs are generated. These TCR Tg mice allow researchers to efficiently study T cell-mediated immune responses according to antigen-specific TCR activity by synchronizing all T cell responses to one epitope [4]. In this review, we focus on the contribution of TCR Tg mice in revealing underlying immunopathological mechanisms of viral infections.

## 2. Importance of T Cells in Viral Immunopathology

Antigen recognition by T cells depends on the physical interaction between TCRs and antigen-major histocompatibility complex (MHC) molecules [5]. TCRs are composed of highly diverse heterodimers and fall into two classes: TCR-αβ and TCR-γδ. TCR α- and γ-chains consist of variable (*V*), joining (*J*), and constant (*C*) regions. The TCR β- and δ-chains consist of *V*, diversity (*D*), *J*, and *C* regions. A V(D)J recombination with an additional nucleotide addition or deletion results in a highly diverse TCR repertoire during T cell development and enables millions of antigens to be recognized. The direct interaction of a TCR with its cognate peptide-MHC complex mediates T cell development, activation, proliferation, differentiation, cytotoxicity, and cytokine production [6,7]. Endogenously synthesized antigens are presented by MHC class I molecules and engage TCRs on CD8^+^ T cells. On the other hand, exogenous antigens in the context of MHC class II molecules are recognized by TCR on CD4^+^ T cells.

An acute infection with virus such as influenza virus is generally accompanied by early production of virions and rapid clearance by a robust host immune system. Although effective anti-viral CD8^+^ and CD4^+^ T cell response generation is critical to rapidly constraining the virus during an acute infection, T cell responses often instead amplify pathogenic outcomes in the presence of sustained viral loads. During chronic infection, like those caused by human immunodeficiency virus (HIV), hepatitis B virus (HBV), and hepatitis C virus (HCV), CD8^+^ T cells become exhausted, and effective memory T cell production is suppressed by persistent antigen stimulation. Additionally, a loss of typical Th1 CD4^+^ T cell function is associated with decreased proliferative and cytokine production potential by chronic antigen exposure [8]. Impaired anti-viral CD8^+^ and CD4^+^ T cell responses lead to elevated chronic viral replication with reduced immunopathogenesis.

The quality and quantity of anti-viral T cell responses are determined by diverse factors such as antigens, antigen-presenting cells, co-stimulatory molecules, and cytokines, which are differentially regulated according to virus type. Thus, TCR Tg mice that can specifically recognize a particular virus are useful in understanding the distinct T cell-mediated immunopathogenesis determined by virus type.

## 3. Generating TCR Tg Mice

Most TCR Tg mice come from an artificially modified genomic construct. The term ‘transgenic’ refers to a genetically modified genome, and the basic principle behind generating TCR Tg mice is based on the mouse germline transformation method [9]. The production of TCR Tg mice starts by generating genomic constructs for transformation. Genes encoding TCR α- or β-chains are extracted from T cell clones expressing antigen-specific TCRs. T cell clones are generated through several methods [10,11] that are followed by the selection of a specific T cell clone. Methods such as sequencing [11], immunofluorescence [12], and cytolytic assays [13] are frequently used to confirm whether the exact Vα or Vβ segment sequences are included in a T cell clone. TCR V(D)J region-specific primers allow recombinant DNA to be inserted into TCR shuttle vectors that include the natural TCRα and TCRβ promoter/enhancer [14]. The genomic construct harboring the rearranged TCR chain is then microinjected into mice with the genetic background of interest. Since engineered TCR genes are inserted into the mouse’s genome early in development, the majority of T cells in Tg mice are monoclonal. However, there are drawbacks to using Tg mice that can hinder efficient research. Some T cell populations may not express a transgene while expressing non-Tg Vα and Vβ TCRs [15,16], even in RAG-deficient mice [17]. Additionally, generating de-novo Tg mice and sequentially backcrossing are time-consuming (6 months to several years) and expensive [4]. To compensate for these disadvantages, more advanced technologies have been introduced. For example, Holst. J et al., 2006 introduced a new model called a retrogenic (‘retro’ from retrovirus and ‘genic’ from transgenic) mouse using retrovirus-mediated stem cell gene transfer [18]. Additionally, a CRISPR-mediated TCR replacement technique can generate TCR Tg mice more efficiently [19].

## 4. Studying Viral Immunopathology with TCR Tg Mice

Diverse TCR Tg mice expressing TCRs specific for viral antigens of LCMV, influenza virus, RSV, WNV, HBV, TMEV, HSV, and MCMV have been generated to contribute to understanding mechanisms of viral immunopathogenesis (Table 1).

### 4.1. Lymphocytic Choriomeningitis (LCMV)

LCMV belongs to the *Arenaviridae* family and has a single-stranded RNA genome [33]. Although mice are its natural host, LCMV infection is a widely used model to study immunopathogenesis caused by viral infection in the animals other than mice. Since LCMV is a non-cytolytic virus, it is possible to accurately measure the cytotoxic activity by the host anti-viral immune response [34]. In addition, it is an advantageous model for studying the pathogenic consequences of infection because several LCMV strains exhibit completely distinct pathological characteristics and display different results depending on the infection route [35]. Importantly, many studies using the LCMV infection model have greatly advanced our understanding of T cell characteristics. For example, mechanisms of MHC-restriction [36,37], perforin-mediated target cell death [38,39], and memory cell generation [40,41,42] that are critical for the generation of T cell-mediated immunopathogenesis were identified using the LCMV infection model.

The most commonly used TCR Tg mouse strain specific to LCMV is the P14 TCR Tg mouse. This mouse expresses TCR specific to the MHC-I-specific LCMV glycoprotein (GP) 33–41 peptide [20]. With an acute strain of LCMV, such as the Armstrong strain, P14 TCR Tg T cells were used to study effector function and memory formation of anti-viral CD8^+^ T cells. For instance, molecular and functional profiling of anti-viral memory CD8^+^ T cells was performed using P14 TCR Tg mice. This led to the discovery of many important memory T cell differentiation-associated molecules such as IL-7 receptor (CD127), KLRG-1, and IL-15 [43,44,45,46]. Additionally, TCR Tg mice were used to identify novel memory subsets such as tissue-resident memory CD8^+^ T cells [47,48]. Interestingly, P14 TCR Tg cells are useful in analyzing the antigen recognition affinity of anti-viral CD8^+^ T cells when analyzed by single cell-based techniques such as the 2D micropipette adhesion frequency assay and bio-membrane force probe assay (BFP) [5,49,50]. Infection with an LCMV chronic strain (Clone 13, Cl13) is frequently used to study mechanisms of viral persistence. Since LCMV Cl13 infection promotes T cell exhaustion, reduces effector properties, and inhibits memory T cell formation [51,52], P14 TCR Tg T mice led to the identification of exhaustion-, effector function-, and memory formation-associated cellular factors such as PD-1, Lag-3, Tim-3, CD160, TIGIT, Tcf-1, sphingosine kinase 2, and Blimp-1 [52,53,54,55,56,57]. These findings could apply to the development of novel therapeutic strategies for diverse human diseases. For example, PD-1, Lag-3, TIGIT, and Tim-3 are upregulated in T cells upon HIV infection [58,59]. Therefore, blockade of these molecules would be applicable as immune checkpoint inhibitor therapy to treat human chronic viral infections.

SMARTA TCR Tg mice express TCR specific for LCMV glycoprotein (GP) 61–80 H2-Ab complexes [21]. Like P14 TCR Tg mice, SMARTA TCR Tg mice helped define CD4^+^ T cell anti-viral mechanisms against acute and chronic LCMV infections. For example, studies using SMARTA TCR Tg mice demonstrated that the TCR signal strength of anti-viral CD4^+^ T cells is critical to memory differentiation and longevity during the primary response [60,61,62]. Additionally, mechanisms that control viral infection via anti-viral CD4^+^ T cells were identified with SMARTA TCR Tg mice [63]. These Tg mice also discerned the role of anti-viral CD4^+^ T cells in rescuing exhausted CD8^+^ T cells during chronic viral infection [54,64,65]. Since CD4^+^ T cell responses are required to generate and maintain protective antibody responses [66], these findings contributed to the enhancement of vaccine efficacy. Additionally, SMARTA TCR Tg mice are useful in comparing distinct anti-viral CD4^+^ T cell responses between acute and chronic viral infections in humans.

### 4.2. Influenza Virus

Despite the existence of anti-viral drugs and vaccines, influenza virus is still a great threat to humans. Influenza viruses cause high morbidity and mortality in immunocompromised individuals and the elderly through respiratory infections [67,68]. Influenza pandemics also cause substantial social and economic problems [68]. Given the importance of T cells in influenza pathogenesis [69], several TCR Tg mice that specifically recognize influenza virus epitopes were developed.

In F5 TCR Tg mice, over 95% of CD8^+^ T cells expressing TCR recognize the influenza A viral nucleoprotein (NP) 366–374 epitope [22]. Studies using F5 TCR Tg mice have revealed important mechanisms involved in effector/memory population generation [70,71] as well as the mechanism facilitating CTL escape during influenza virus infection [72]. Thus, F5 TCR Tg mice have aided in vaccine design against viruses that undergo frequent mutation. Clone-4 (CL4) TCR Tg mice express TCR specific for the HA peptide epitope IYSTVASSL of the PR8 strain of influenza virus [23]. Morgan et al. used this TCR Tg mouse strain to investigate the onset of peripheral tolerance induction upon influenza virus infection. They showed that the lack of tolerance during the perinatal period correlated with a lack of antigen-specific CD8^+^ T cell activation, providing evidence for the induction of peripheral tolerance [73]. Because CD8^+^ T cell-mediated immunopathogenesis is a central mechanism to eliminate virus-infected target cells, these studies contribute to the development of more effective T cell-mediated therapy against influenza virus infections.

Since CD4^+^ T cells orchestrate anti-influenza viral immune responses including the CTL response and antibody generation [74], understanding CD4^+^ T cell mechanisms is critical for developing novel influenza vaccines. TS1 mice bear TCR specific for the HA peptide 111–119 presented in MHC-II I-Ed [24]. These mice have been used to discern how CD4^+^ T cells specifically contribute to effector immune cell generation, inflammatory cytokine production, and effector cell recruitment into the lungs during influenza virus infection [71,75].

Therefore, understanding of influenza virus infection-mediated CD8^+^ and CD4^+^ T cell response and pathogenesis using these TCR Tg mouse models would lead to the development of more efficient therapeutics and vaccines for human influenza infection.

### 4.3. Respiratory Syncytial Virus (RSV)

Respiratory Syncytial Virus (RSV) is an enveloped, single-stranded RNA virus that causes lower respiratory tract infections especially in infants and young children [76]. Mice have been used extensively as an RSV animal infection model, and they have provided insights into immunological and pathological mechanisms of RSV infection. Two CD8^+^ TCR Tg strains (TRBV13-1 and TRBV13-2) both specific to the RSV-dominant KdM282-90 epitope were generated by Bar-Haim and colleagues [25]. By using these TCR Tg mice, the differential TCR repertoire between RSV infection and vaccination was investigated [77]. Additionally, these mice helped identify the role of neonatal CD103^+^ dendritic cells (DCs) in generating a distinct anti-viral CD8^+^ T cell response separate from that of adults [78]. Thus, these results would contribute to a better understanding of T cell function and immunopathogenesis against RSV infection in humans. In addition, studies with TCR Tg mice for RSV provide insights into the development of more efficient neonatal and infant vaccines. 

### 4.4. West Nile Virus (WNV)

West Nile virus (WNV), a positive-sense single-stranded RNA virus that belongs to the *Flaviviridae* family, is a neurotropic virus. WNV is transmitted to humans via mosquitos and causes significant global public health problems [79]. TCR Tg mice were generated by Kim and colleagues to study WNV (WNV-I mice) [26]. This TCR Tg mouse contains TCRs specific for the immunodominant epitope of the WNV NS4B protein. By using WNV-I TCR Tg mice, the kinetics, expansions, and differentiation of WNV-specific CD8^+^ T cells within the spleen and brain were identified. Importantly, the critical role of inflammatory cytokines and the ability of tissue-resident anti-viral CD8^+^ T cells in reducing viral burden within the brain was demonstrated using WNV-I TCR Tg mice [80]. Thus, this TCR Tg mouse model could be a novel resource for understanding the protective and immunopathological mechanisms of anti-viral CD8^+^ T cells against WNV infection in humans.

### 4.5. Hepatitis B Virus (HBV)

Hepatitis B virus (HBV), a member of the *Hepadnaviridae* family, is a noncytopathic enveloped virus with a circular double-stranded DNA genome. It can cause acute and chronic hepatitis and hepatocellular carcinoma [81,82]. Although vaccines against HBV are available, chronic hepatitis caused by HBV still leads to a significant number of deaths [83]. During chronic HBV infection, the functionality and dynamics of antiviral immune cells are impaired and altered; this includes changes in cytokine production [81,82]. Chen and colleagues introduced HBV Tg mice (11/4-12), which have nucleoprotein HBeAg-specific CD4^+^ T cells. This model showed that HBeAg-specific CD4^+^ T cells could be activated by exogenous HBeAg and elicit liver injury, suggesting the pathological role of HBV nucleoprotein-specific CD4^+^ T cells in liver injury [27]. Other HBV-specific TCR Tg mice (BC10.3 and 6C2) that target the nucleocapsid (COR93 epitope) and envelope (ENV28 epitope) proteins of HBV were generated by Isogawa and colleagues in 2013. These models provided insight into how the activation of HBV-specific CD8^+^ T cells is regulated by the balance between PD-1 inhibitory and stimulatory signals against intrahepatically expressed HBV [28]. Thus, the development of HBV-specific TCR Tg mice models can shed light on the complex interactions between T cells and other immune cells during HBV infection. Moreover, the HBV-specific TCR Tg mouse model could help to identify underlying mechanisms of how CD8^+^ T cell exhaustion is caused by HBV, which can lead to the development of therapy for chronic viral infection and perhaps other persistent viral infections as well.

### 4.6. Theiler’s Murine Encephalomyelitis Virus (TMEV)

Theiler’s murine encephalomyelitis virus (TMEV) was discovered in 1933 and caused encephalomyelitis and paralysis in mice [84]. Because of its persistent infection of the central nervous system and demyelinating, TMEV has been used as a mouse model to study paralysis associated with viral infection, encephalomyelitis, and multiple sclerosis [85]. TCR Tg mice (VP2) that specifically recognize TMEV capsid protein VP274-86 were used to identify the novel pathogenic role of Th17 T cells in persistent viral infection [29]. Since Th17 cells are one of the essential immune cells implicated in the pathogenesis of neuroinflammatory diseases such as acute disseminated encephalomyelitis and multiple sclerosis in humans [86], this TCR Tg mouse model would be useful to unveil the underlying mechanism for disease progression of neuroinflammatory disorders. 

### 4.7. Herpes Simplex Virus (HSV)

Herpes simplex virus (HSV) is a member of the *Herpesviridae* family. It is a highly contagious, recurrent, life-long, and double-stranded DNA virus. Type 1 HSV (HSV-1) is mainly transmitted via oral-to-oral contact, but HSV-2 is sexually transmitted [87]. Since severe HSV-1 symptoms are exhibited in immune-comprised patients, understanding the immunopathogenesis during HSV infection is critical for developing anti-viral therapies. In 2002, two strains (gBT-I.1 and gBT-I.3) of HSV-1 specific TCR Tg mice (H-2Kb-restricted HSV-1 glycoprotein B498-505) were generated by Mueller and colleagues [30]. These Tg mice provided insights into the complex mechanisms of how anti-viral CD8^+^ T cells display protective function to control HSV-1 latency and corneal neovascularization provoked by HSV-1. Additionally, a study using these mice demonstrated that TLR3 activation in DCs could generate more robust anti-viral CD8^+^ T cell responses, which can apply to the development of novel vaccine adjuvants. [88,89,90]. Thus, the elucidation of HSV-specific CD8^+^ T cell dynamics is expected to accelerate the development of improved therapeutic strategies that ultimately reduce HSV infection-induced lethality.

### 4.8. Murine Cytomegalovirus (MCMV)

Murine cytomegalovirus (MCMV) is a rodent pathogenic virus that causes life-long latent infection [91]. Two TCR Tg mouse strains (M38 and M25) have been used to study T cell-mediated immunopathogenesis against MCMV infection. The M38 TCR Tg mouse expresses the MHC-I-restricted MCMV epitope M38-specific TCR [31]. Studies using this mouse have helped characterize the inflationary CD8^+^ T cell response mechanism during MCMV infection [31,92]. The M25 TCR Tg mouse specifically recognizes the MHC-II-restricted MCMV epitope M25 [32]. This strain allowed researchers to study the functional characteristics of MCMV-specific CD4^+^ T cells and the roles of cytokines IL-10 and IFN-γ in regulating anti-viral CD4^+^ T cell-mediated pathogeneses [32,93,94]. Since the MCMV mouse infection model has been used to understand pathogenesis caused by human cytomegalovirus (HCMV) infection, studies with these TCR Tg mice would improve our understanding of T cell-mediated immune response and pathogenesis to develop the vaccine for HCMV.

## 5. Recombinant Viruses Bearing Epitopes to Common TCR Tg Mice 

When TCR Tg mice to specific viruses are unavailable, recombinant viruses encoding immunodominant epitopes of commonly available TCR Tg mice can be used as an alternative. With this system, researchers can overcome the limitations of traditional methods and easily assess analytical tools that are already established. Several studies have utilized OVA-specific OT-I or OT-II TCR Tg mice to study viral pathogenesis generated by diverse viruses because OT-I and OT-II TCR Tg mice are the most commonly available Tg mice. For example, influenza viruses (A/WSN/33 or A/PR8/1934) expressing the partner antigen (OVA) were generated to utilize OT-I and OT-II TCR Tg mice. These mice identified roles of viral antigen-specific CD4^+^ and CD8^+^ T cells in host immune responses [95,96,97,98]. In addition to studying the characteristics of T cell-mediated viral pathogenesis, other recombinant viruses including HSV [99], murine gammaherpesvirus 68 (MHV-68) [100], and vaccinia virus (VACV) [101,102] have been used in combination with OT-I or OT-II TCR Tg mice. LCMV antigen-specific P14 and SMARTA are also conveniently available TCR Tg mice. Thus, many studies have utilized recombinant viruses expressing LCMV antigen, including influenza virus and VACV, along with P14 and SMARTA TCR Tg mice [21,103,104,105,106] to identify the roles of anti-viral CD4^+^ and CD8^+^ T cells in generating memory responses. Thus, the use of recombinant viruses that express antigens recognized by common TCR Tg mice is a useful alternative strategy to investigate viral pathogenesis.

## 6. Conclusions and Discussion

This review was designed to provide background knowledge about TCR Tg mice and summarize their various applications in studying T cell immunopathogenic mechanisms against viral infections. The high repertoire diversity is important for hosts to recognize various foreign antigens; however, it impedes the investigations of CD4^+^ and CD8^+^ T cell responses that are central to eliminating and preventing reinfections. Diverse TCR Tg mice expressing TCRs specific for viral antigens can overcome the limitations mentioned above. By using TCR Tg mice, researchers have uncovered novel mechanisms of viral infection and spurred advancements in antiviral therapeutic and vaccine development.

While TCR Tg mice are a useful tool to study antigen-specific T cells, there are some limitations. For example, since humans are continuously exposed to a large number of various pathogens, TCR Tg mice that are manipulated to react to a single epitope cannot fully reflect the complex pathogeneses of human diseases. Nevertheless, to rapidly respond to newly emerged viruses such as coronavirus and influenza virus variants, understanding exactly how T cell responses are regulated according to the type of antigen is essential. Thus, the generation of TCR Tg mice specific for various viruses would be in great demand to develop more efficient therapy for viral pathogenesis. Ongoing progress in the genetic manipulation and sequencing fields will promote the generation of more diverse TCR Tg mice in more efficient ways.

Although this review mainly focused on the importance of TCR Tg mice in exploring infectious viral diseases, TCR Tg mice are also invaluable tools for understanding the pathogenic roles of T cells in immune-associated diseases including cancers, autoimmune disease, and infectious bacterial diseases.

## Figures and Tables

**Table 1:** TCR Transgenic mice used in the viral immunopathological studies.

Target Virus	Name	CD4/CD8	Epitope	Positions	Ref.
LCMV	P14	CD8	KAVYNFATM	Glycoprotein 33–41	[20]
	SMARTA	CD4	GLKGPDIYKGVYQFKSVEFD	Glycoprotein 61–80	[21]
Influenza virus	F5	CD8	ASNENMDAM	Nucleoprotein 366–374 (A/NT/60/68)	[22]
	Clone-4	CD8	IYSTVASSL	Hemagglutinin 533–541 (strain A/PR8/1934)	[23]
	TS1	CD4	SFERFEIFPK	Hemagglutinin 111–119 (strain A/PR8/1934)	[24]
RSV	TRBV13-1	CD8	SYIGSINNI	M2 protein 82–90	[25]
	TRBV13-2	CD8	SYIGSINNI	M2 protein 82–90	[25]
WNV	WNV-I	CD8	SSVWNATTA	NS4B 2488–2497	[26]
HBV	11/4-12	CD4	PPAYRPPNAPIL	Nucleoprotein HBeAg 129–140	[27]
	BC10.3	CD8	MGLKFRQL	Core protein 93–100	[28]
	6C2	CD8	IPQSLDSWWTSL	Envelope 28–39	[28]
TMEV	VP2	CD4	QEAFSHIRIPLPH	Capsid protein VP 274–286	[29]
HSV	gBT-I.1	CD8	SSIEFARL	Glycoprotein B 498–505	[30]
	gBT-I.3	CD8	SSIEFARL	Glycoprotein B 498–505	[30]
MCMV	M38	CD8	SSPPMFRV	M38 protein 316–323	[31]
	M25	CD4	NHLYETPISATAMVI	M25 protein 409–423	[32]
